# Proximal Tubular Deposits and an Abnormal MRI of the Kidneys with Chronic Hemolytic Anemia

**DOI:** 10.34067/KID.0000000000000086

**Published:** 2023-05-25

**Authors:** Takumi Toishi, Tomo Suzuki

**Affiliations:** Department of Nephrology, Kameda Medical Center, Chiba, Japan

**Keywords:** glomerular and tubulointerstitial diseases, hemolytic anemia, hemosiderosis, magnetic resonance imaging, kidney biopsy

## Abstract

**Figure:**
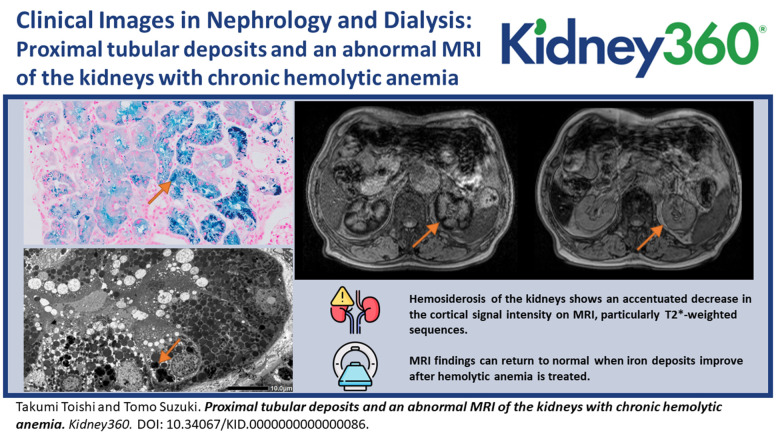

## Case Description

**Figure 1:**
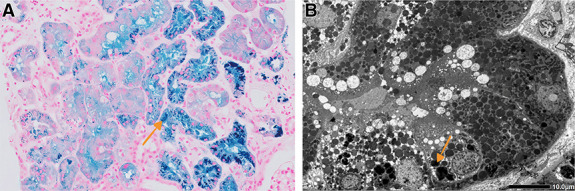
**Kidney biopsy.** Light microscopy of the second kidney biopsy shows Berlin blue staining positivity of the granules in the proximal tubules (A). The orange arrow points at granules indicating iron deposition. Electron microscopy shows an increase in lysosomes in the tubules and lysosomal iron phagocytosis (B). The orange arrow points at iron in lysosomes.

**Figure 2:**
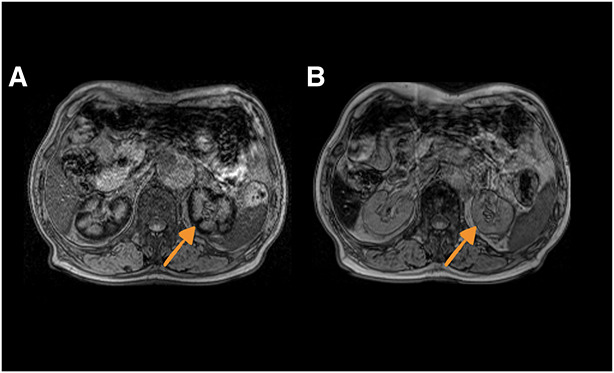
Magnetic resonance imaging shows changes in renal cortical signal intensity on T2*-weighted sequences. The images on the left (A) and right (B) were obtained before and after the repeat surgery, respectively. The left image shows an accentuated decrease in the cortical signal intensity on T2*-weighted sequences. The right image shows a diminishing signal change.

A 66-year-old man with traumatic hemolytic anemia due to a vascular graft for type A aortic dissection had developed hematuria and nephrotic syndrome 3 years earlier. His blood and urine test results indicated nephrotic syndrome (serum creatinine, 0.99 mg/dl; serum albumin, 2.6 g/dl; and protein/creatinine ratio in urine, 15.1 g/gCr). In the first kidney biopsy, light microscopy showed minor glomerular changes with dark brown granules in the proximal tubules. Electron microscopy did not reveal diffuse foot process effacement. Unfortunately, immunofluorescence stains could not be interpreted because of their poor quality. After initiating prednisolone treatment, the urinary findings improved. The hemolytic anemia also partially improved. His proteinuria worsened with declining kidney function (serum creatinine, 1.56 mg/dl; serum albumin, 3.3 g/dl; and protein/creatinine ration in urine, 10.6 g/gCr) 2 years earlier. A second kidney biopsy was performed. Light microscopy revealed an increase in dark brown granules in the proximal tubules; however, there was no mesangial and endothelial proliferation in the glomerulus. Berlin blue staining was positive for the granules, which indicated iron deposits (Figure 1A). The tubules with granules had atrophied slightly. Immunofluorescence stain revealed negative results for IgG, IgA, IgM, C3, and C1q. Electron microscopy showed an increase in lysosomes in the tubules and lysosomal iron phagocytosis (Figure 1B). Kidney histology was consistent with kidney hemosiderosis. We used magnetic resonance imaging (MRI) to assess iron deposition. T2-weighted imaging showed an accentuated decrease in cortical signal intensity, particularly for the T2*-weighted sequences. (Figure 2) Therefore, we diagnosed renal hemosiderosis and repeated surgery to treat hemolytic anemia and declining kidney function. During surgery, the inverted felt material of the aortic root was removed. Hemolytic anemia improved after surgery, and kidney function stabilized. The initial MRI abnormality was also not present on T2-weighted imaging and sequences (Figure 2).

## Discussion

Hemosiderosis of the kidney is caused by traumatic hemolytic anemia or paroxysmal nocturnal hemoglobinuria.^1^ Kidney histology is characterized by iron deposition in proximal tubules. Excessive hemolysis begins with the liberation of hemoglobin into the plasma, which is filtered through the glomerulus and is reabsorbed along the proximal tubules by the megalin-cubulin receptor, resulting in iron deposition.^2^ Several mechanisms underlying hemosiderosis of the kidney have been postulated. They include decreased kidney perfusion due to vasoconstriction caused by nitric oxide sequestration, direct cytotoxicity to proximal tubules, and tubular obstruction by hemoglobin casts.^3^ In this patient, kidney biopsy revealed iron deposits in the proximal and atrophied tubules with granules, but few casts in the tubule lumen. As with this patient, the usefulness of MRI, especially T2*-weighted sequences, has been reported.^4^ We confirmed improvements in the abnormal findings by performing MRI before and after the corrective surgery. MRI is not often used to diagnose hemosiderosis of the kidney. We believe these findings are important but have not been previously reported.

In conclusion, we diagnosed hemosiderosis of the kidney based on MRI and kidney biopsy. MRI may be useful in diagnosing hemosiderosis of the kidney.

## Teaching Points

- Hemosiderosis of the kidney shows an accentuated decrease in cortical signal intensity on T2-weighted MRI, particularly T2*-weighted sequences.
- MRI findings can return to normal when iron deposits improve after hemolytic anemia is treated.
